# The Schistosomiasis Research Agenda—What Now?

**DOI:** 10.1371/journal.pntd.0000207

**Published:** 2008-02-27

**Authors:** Jeffrey M. Bethony, Alex Loukas

**Affiliations:** 1 Department of Microbiology, Immunology and Tropical Medicine, George Washington University, Washington, D.C., United States; 2 Division of Immunology and Infectious Diseases, Queensland Institute of Medical Research, Queensland, Australia; Swiss Tropical Institute, Switzerland

## A Schistosomiasis Research Agenda

As relatively new schistosomiasis researchers, we awaited with eagerness the publication of the “Schistosomiasis Research Agenda” (SRA) put forward by Colley and Secor in the December 2007 issue of *PLoS Neglected Tropical Diseases* [1]. The SRA is a comprehensive, well-organized list of research activities that reflects the impressive diversity of interests that make up current schistosomiasis research. Colley and Secor went to admirable lengths to solicit the interests of researchers the world over, with special efforts to solicit the opinions of scientists in countries or regions where schistosomiasis is endemic, such as Brazil, China, and Africa. Having attended some of these meetings (11th International Congress of Parasitology, held in Glasgow, United Kingdom in August 2006; and the 55th Annual Meeting of the American Society of Tropical Medicine and Hygiene, held in Atlanta, United States in November 2006) and received the e-mails, we are confident that the SRA indeed reflects the richness and breadth of current schistosomiasis research.

As noted by Colley and Secor [1], many of these areas of interest in the SRA are applicable to the study of almost any neglected tropical disease (NTD). However, while research into other tropical diseases such as malaria and a number of the NTDs—most notably hookworm disease, cysticercosis, and leishmaniasis—are currently enjoying a “renaissance”, with increased funding from major philanthropies such as the Bill and Melinda Gates Foundation [2], research into schistosomiasis remains one of the truly neglected areas of NTDs. This problem exists despite the fact that schistosomiasis is arguably the most important human helminth infection in terms of global morbidity and mortality as measured by disability-adjusted life-years (DALYs). Recently, King et al. [3] revised upwardly the DALY estimates for schistosomiasis, by including not only gross organ pathology as a disability, but also the anemia, pain, diarrhea, exercise intolerance, and under-nutrition that result from chronic infection with schistosomes. In 2003, the Gates Foundation provided a grant of US$30 million to create the Schistosomiasis Control Initiative (SCI), an organization that facilitates mass administration of praziquantel (PZQ) currently in six African countries [4]. The use of PZQ as a safe, inexpensive, and efficacious method to resolve current schistosomiasis infection and morbidity is admirable; however, there has developed an unexpected, yet serious, long-term side effect—the spurious perception that widespread use of PZQ makes schistosomiasis a problem of the past [5]. This misconception has promoted the belief amongst some funding bodies that we already have all the requisite tools to control schistosomiasis (i.e., PZQ), and development of new control strategies is unnecessary. Given the extensive burden of disease related to schistosomiasis, relying solely on mass and repeated treatment of exposed populations with PZQ is not enough to sufficiently control, let alone eradicate, this disease [6],[7].

## Diversity versus Divisiveness

As noted by Colley and Secor [1], the diversity of backgrounds and interests in schistosomiasis, while enriching the field, may have also led to a “divisiveness” that has harmed its progress. In our opinion, there has been no greater area of divisiveness in schistosomiasis research than the debate on the use of chemotherapy versus vaccines for controlling schistosomiasis [8]–[12]. The debate did not result in a “fruitful reorientation of schistosomiasis research” as proferred [8], but has solidified researchers into the simplistic camps of “for” and “against” vaccines [7]. Furthermore, although we agree that there is much diversity in the field of schistosomiasis research, we do not feel that this diversity is inherently harmful. Perhaps even more troubling is the chronic discord within disciplines, whether it is epidemiology, immunology, genomics, proteomics, or control.

## A Way Forward

Rather than commenting on the exhaustive list of interests spanned and the numerous combinations of research interests and disciplines possible, we have instead chosen to discuss mechanisms by which the diverse interests of the SRA might be integrated into a potential way forward for the field. We feel that this is best accomplished by looking outside of schistosomiasis to fields in which similar diversities—but not divisiveness—exist and researchers work harmoniously and productively. Box 1 highlights some examples of networks that are considered to be highly successful by many of their respective members. For instance, malaria research is a large and highly competitive field, but a number of networks and foundations exist to foster collaboration, communication, and interactions amongst members. This is best exemplified by BioMalPar, which has been a great success for the malaria community and laboratories in both Europe and endemic countries. Many consider the flagship of BioMalPar to be its PhD program, which is centered on joint supervision of doctoral students and genuine time spent in multiple laboratories in different countries. Their scientific conferences and the degree of openness amongst malaria groups (many of which were traditionally rivals) are considered to be truly impressive by malariologists.

We have interacted with the other two networks listed in Box 1, which we believe are equally successive at bringing researchers together.

**Box 1. Some Models of Interdisciplinary Research Networks**

1. BioMalPar – “Biology and Pathology of Malaria Parasite” is a Network of excellence funded by the European Commission. Thirty-two leading institutes from 10 European and 6 developing countries involved in fundamental research coordinate their efforts in a virtual, multi-center “European Malaria Research Institute”. Many consider their flagship to be a PhD program that supports collaborative research projects between two or more institutions. http://www.biomalpar.org/
2. ARC/NHMRC Parasitology Research Network – Australian government funded network to promote collaborations between Australian and international researchers. Funding for collaborative travel and grant writing retreats and an annual conference are provided by the network. http://parasite.org.au/arcnet/
3. Consortium for Functional Glycomics – a large research initiative, funded by the National Institutes of Health (NIH), and formed to define the paradigms by which protein-carbohydrate interactions mediate cell communication. The strategy is to work with the scientific community to create unique resources and services that participating investigators can utilize in their own research. http://www.functionalglycomics.org/static/index.shtml

## Why Do These Networks Work?

The successful networks highlighted above have one thing in common: they are well funded. However, this was not always the case—these researchers had to come together, agree on a granting agency to target, and develop a suitable agenda by which to solicit funding. We suggest that the SRA is the place to start a similar effort for schistosomiasis with the following objectives: (a) fostering interdisciplinary methods; (b) standardizing research protocols; (c) elevating the profile of schistosomiasis within the global health community; (d) creating repositories of biomaterial; and (e) utilizing expertise outside of schistosomiasis. An example of a well-funded cooperation within schistosomiasis already exists. The Biomedical Research Institute (BRI) in Maryland, US is a facility that supports schistosomiasis research through the provision of parasite material and a repository for reagents. The BRI schistosomiasis program is funded by National Institutes of Health, highlighting to the community that granting bodies are prepared to fund schistosomiasis research and nurture collaborative efforts.

## A Start

We need to build upon the momentum created by the SRA. As a start, we should not consider the SRA as a static document, nor the end of a process, but the start of one. Indeed, one of the best aspects of the SRA was the transparent manner in which it was created and composed, which involved an extensive emailing list, frank conversations between researchers, lively meetings of the schistosomiasis community at major conferences, and the understanding that the SRA would not promote any one group of researchers or area of research, but was a voice for the entire community. With this infrastructure already in place, we should come together in an effort to secure funding that does not directly benefit any one of our research programs, but further unifies the community, accelerating its “recovery” to that warranted by the severity of the disease itself.
